# Editorial: Multi-site neuroimage analysis: Domain adaptation and batch effects

**DOI:** 10.3389/fninf.2022.994463

**Published:** 2022-08-31

**Authors:** Muhammad Yousefnezhad, Daoqiang Zhang, Andrew J. Greenshaw, Russell Greiner

**Affiliations:** ^1^Department of Computing Science, University of Alberta, Edmonton, AB, Canada; ^2^Department of Psychiatry, University of Alberta, Edmonton, AB, Canada; ^3^Alberta Machine Intelligence Institute (Amii), Edmonton, AB, Canada; ^4^Department of Computer Science and Technology, Nanjing University of Aeronautics and Astronautics, Nanjing, China; ^5^Canadian Institute for Advanced Research (CIFAR) AI Chair, Toronto, ON, Canada

**Keywords:** multi-site neuroimage analysis, domain adaptation, batch effects, transfer learning, machine learning

Neuroimaging provides a vital tool for both theoretical and empirical studies of human brains, including the investigation of cognitive processes and neurodevelopmental trends, and the assessment and diagnosis of brain disorders (Yousefnezhad et al., 2020; Zhou et al., 2020). Despite the advantages of modern imaging technologies, this is an extremely challenging task as the data are high dimensional and noisy, and most datasets are based on relatively few scans (partly due to the expense of neuroimage acquisition) (Yousefnezhad et al., 2020). As finding subtle patterns often requires larger sample sizes, researchers are investigating ways to combine data from many sites. The obvious approach is building a large dataset formed by simply concatenating data from numerous sites/sources; unfortunately learning a model from such a dataset is often problematic—in particular, less accurate than a model trained on a single site (Zhang et al., 2018). This may be due to batch effects or covariate shifts, etc. Domain adaptation techniques—which use domain knowledge from the source datasets to improve the performance of related target data—hold great promise for addressing these issues (Zhang et al., 2018; Yousefnezhad et al., 2020; Zhou et al., 2020).

In this Research Topic, we have collected 7 research studies that describe ways to apply advanced machine learning approaches to address current challenges in multi-site neuroimaging analyses. Several of these studies address homogeneous domain adaptation issues, where both the source and target sites used the same modularity in their neuroimaging data—e.g., both comprised fMRI data based on the same tasks. Other studies dealt with non-homogeneous problems, where the source and target sites have different image modalities—e.g., MRI and fMRI. The following paragraphs describe the principal contributions of each of these studies.

Panda et al. introduced the Multi-source Domain Adversarial Networks (MSDA) approach that uses latent features for learning an effective classification model. They showed that their proposed approach can produce accurate predictions in comparison with various MSDA approaches—including Multi-source Domain Adversarial Networks (MDAN), Domain AggRegation Networks (DARN), Multi-Domain Matching Networks (MDMN), and Moment Matching for MSDA (M3SDA)—for predicting specific labels (e.g., illness, age, or sex) using resting-state fMRI responses from two publicly available datasets: ABIDE 1 and ADHD-200.

Invariant Information Clustering (IIC) is a novel deep neural network model that extracts invariant information from multi-source resting-state fMRI datasets. Okamoto and Akama proposed Extended IIC (EIIC), which uses contrastive learning that is characterized by transfer learning with labeled data pairs but without the need for a data augmentation technique. They applied their EIIC to the resting-state fMRI dataset provided by the Autism Brain Imaging Data Exchange, and demonstrated better classification accuracy than other classification methods, for the majority of scanned locations in autism spectrum disorder (ASD).

Bento et al. studied brain MRI datasets incorporating data from control subjects and from patients with various diseases (from psychiatry, neurology cancer, etc.), from 17 publicly available data repositories—first discussing Data Access issues, then outlining the key characteristics of these commonly used publicly available brain datasets. Subsequently, they reviewed two main approaches for correcting batch effects: (1) Data Harmonization, which uses data standardization, quality control protocols, and other similar algorithms and processes to understand and minimize unwanted batch effects; and (2) Domain Adaptation, which develops DL tools that handle batch effects implicitly through approaches for achieving reliable and robust results. Their empirical results show that these approaches produced better results, over the baseline (of no such corrections).

Choi et al. investigated brain aging in East Asian people by measuring cortical and subcortical volumes from magnetic resonance imaging (MRI) scans of 1,008 cognitively normal elderly Koreans from the Gwangju Alzheimer's and Related Dementia cohort and 342 Caucasians from the Alzheimer's Disease Neuroimaging Initiative (ADNI) database. This study used beta coefficients of age and confidence intervals (CIs) to examine whether aging effects differ with ethnicity and sex. The authors report that most of the ethnic differences observed among females may be explained by synergistic effects between ethnic background and *APOE ε*4 carrier status.

Liu et al. developed a deep framework to reconstruct neonatal white matter (WM) and pial surfaces. In the proposed method, T1-weighted MRI images of neonatal brain tissues are used to address the mis-segmentation of brain tissues. Moreover, this pipeline improves cortical boundary detection by combining Cerebro-Spinal Fluid (CSF) and Gray Matter (GM) boundary detection models with edge gradient information and skeletonizing the foldings of the sulci, where no CSF voxels can be seen due to the limited resolution. The landmark-based evaluation revealed that mean displacement of the cortical surfaces from the true boundaries was less than a voxel size (0.532±0.035 mm). Evaluating the proposed pipeline also demonstrated robustness and reproducibility across different sites and different age groups.

Iyer et al. examined the ability of spiking neural networks (SNNs) to use spike timings in calculations when using neuromorphic datasets recorded from static images. The authors explored SNN algorithms using three hypotheses that examined: (1) whether additional information in neuromorphic datasets is encoded in the time domain or not; (2) two Spike Timing Dependent Plasticity (STDP) algorithms to show that these methods can only classify spatial data; and (3) whether STDP-tempotron approaches can also classify spatiotemporal data. The results of this paper lead to an open question: what dataset may be used to benchmark SNN's ability to classify temporal data?

Rasgado-Toledo et al. introduced a novel dataset to study pragmatic language and its underlying cognitive process. The study involved 145 neurotypical volunteers who were Mexican-born (79 females and 66 males) and who spoke Spanish as their native language, aged 17–35 years and with a range of 12–22 years of education. No psychological distress or psychiatric disorders have been reported for these subjects using the Spanish version of Symptom Checklist 90 (SCL-90, mean 0.63, SD 0.46).

The results presented in these excellent papers address an impressive range of approaches for several challenges in analyzing multi-site brain data using domain adaptation and transfer learning.

## Author contributions

MY wrote the first draft of this report, and other authors edited and revised the article. All authors contributed to the article and approved the submitted version.

## Conflict of interest

The authors declare that the research was conducted in the absence of any commercial or financial relationships that could be construed as a potential conflict of interest.

## Publisher's note

All claims expressed in this article are solely those of the authors and do not necessarily represent those of their affiliated organizations, or those of the publisher, the editors and the reviewers. Any product that may be evaluated in this article, or claim that may be made by its manufacturer, is not guaranteed or endorsed by the publisher.
